# Suppression of focal adhesion formation may account for the suppression of cell migration, invasion and growth of non-small cell lung cancer cells following treatment with polyisoprenylated cysteinyl amide inhibitors

**DOI:** 10.18632/oncotarget.25372

**Published:** 2018-05-25

**Authors:** Elizabeth Ntantie, Michaela J. Allen, Jerrine Fletcher, Augustine T. Nkembo, Nazarius S. Lamango, Offiong F. Ikpatt

**Affiliations:** ^1^ College of Pharmacy and Pharmaceutical Sciences, Florida A&M University, Tallahassee, FL 32307, USA; ^2^ Department of Pathology, University of Miami, Coral Gables, FL 33027, USA

**Keywords:** focal adhesion, integrins, fascin, metastasis

## Abstract

Migratory cells form extracellular matrix attachments called focal-adhesions. Focal adhesion assembly and disassembly are regulated by the Rho family of small GTPases. We previously reported that polyisoprenylated cysteinyl amide inhibitors (PCAIs) suppress Rho protein levels, disrupting F-actin cytoskeleton remodeling in the formation of lamellipodia and filopodia. In this study, we investigated whether these observations effect focal adhesion formation, which involves cell surface receptors known as integrins and several signaling/adaptor proteins such as vinculin, α-actinin, Rock kinases and phospho-Myosin Light Chain-2 (p-MLC-2), that foster the linkage of the actin cytoskeleton to the extracellular matrix. We observed that treatment of H1299 cells with 5 μM PCAIs for 24 h markedly diminished the level of full-length integrin α4 by at least 24% relative to controls. PCAIs at 5 μM, diminished the levels of vinculin by at least 50%. Immunofluorescent analysis showed at least a 76% decrease in the number of vinculin-focal adhesion punctates. In addition, PCAIs diminished Rock1 levels by 25% and its substrate, p-MLC-2 by 75%. PCAIs did not significantly alter the levels of integrin β5, α-actinin, and Rock2, suggesting that the effects of the PCAIs are target specific. Our data indicate that the PCAIs alter the levels of the Rho proteins and their effectors to abrogate their functions in cytoskeleton remodeling thereby suppressing focal adhesion formation. This in turn results in a PCAIs-induced decrease in cell invasion, thus making the PCAIs propitious agents for the inhibition of cancer growth and metastasis.

## INTRODUCTION

Tumor metastasis causes significant morbidity and mortality related to involvement of distant organs from primary or initial organ of involvement [1]. The process of metastasis requires adhesion of the migrating tumor cells to secondary sites where they colonize the new sites. Cell migration and adhesion are well orchestrated dynamic cellular processes that involve actin cytoskeleton remodeling. Dysregulation of these processes enhances metastasis [1].

The adhesion of cells to extracellular matrix (ECM) proteins results in the formation of cell-matrix contact points. These are known as focal adhesions. Focal adhesions allow the interaction of cells with their environment. Focal adhesion plays important roles in embryonic development, maintenance of tissue integrity and organ functions [2]. They have structural and signaling roles through the linking of the ECM to the actin cytoskeleton by generating traction forces necessary for cell migration and its regulation [3]. Proteins that are involved in focal adhesion can be categorized into membrane proteins (such as integrins which connect the ECM to the actin cytoskeleton), adapter proteins (such as vinculin and alpha-actinin that bind actin and connect it to the integrins), and signaling proteins (such as the Rock kinases that mediate adhesion assembly and disassembly) [2].

Integrins, expressed in almost every cell type, are cell surface heterodimeric receptors for ECM glycoproteins [4–6]. They consist of non-covalently linked α and β subunits. Each subunit has a large extracellular domain that binds ECM, a single membrane-spanning domain, and a short non-catalytic cytoplasmic tail, important for protein-protein interactions. The cytoplasmic tail interacts with actin-binding proteins (α-actinin and vinculin) that connect integrins to the cytoskeleton. These actin-binding proteins localize to focal adhesions and strengthen focal adhesion formation and turnover [7]. By connecting integrins to the actin cytoskeleton, vinculin and α-actinin mediate the transmission of contractile forces and signals between the ECM and the cytoskeleton. It is thus not surprising that depletion of vinculin has been reported to disrupt cell adhesion while promoting apoptosis [8].

The role of integrins in maintaining stable adhesion and promoting cell migration is largely dependent on its connection to the F-actin cytoskeleton. Metastatic cells alter the levels of integrin expression and integrin affinity for ECM substrates [9–12]. Inducing αv subunit or β3 subunit expression in melanoma was reported to increase their metastatic potential [12]. Integrin α6β4 not expressed in normal thyroid cells, was reported to be expressed in invasive thyroid carcinoma [13] and in papilloma [14]. In some cases, integrin expression has been reported to decrease during tumorigenesis. For example, decreased expression of α1, α6, β1 and β4 integrin subunits have been documented in breast cancer [15].

Integrin-mediated formation of focal adhesion is regulated by RhoA, a member of the Rho family of small GTPases. RhoA activates signaling molecules such as the Rho-kinases (Rock1 and Rock2) which phosphorylate the actomyosin cytoskeleton, myosin light chain of myosin-2, to generate phospho-myosin light chain-2 (pMLC-2) that organize with the F-actin cytoskeleton-linked to integrins to induce the assembly of focal adhesions [16].

Pharmacological agents that modulate the levels of any of the aforementioned proteins; RhoA, integrins, α-actinin, vinculin, p-MLC-2, Rock1 or Rock2 are likely to affect the assembly and/or disassembly of focal adhesions and thus alter cell motility. Such agents would be of great interest, as effective therapies for the treatment of metastatic lung cancer and other cancers are limited and the prognosis for the metastatic non-small cell lung cancer disease remains poor with a 5 year survival rate of 1% [17].

We recently reported that a novel class of compounds (PCAIs), induced a decrease in the levels of RhoA in the NCI-H1299 non-small cell lung cancer cells to disrupt F-actin organization and abrogate cell migration [18]. Here, we examine the effects of the PCAIs on focal adhesion by investigating their effects on focal adhesion proteins including integrins, α-actinin, vinculin, Rock1, Rock2 kinases, pMLC-2. We report that PCAIs selectively deplete the levels of integrin α4 subunit, vinculin, Rock1 and its substrate p-MLC-2. PCAIs did not significantly alter the levels of integrin β5 subunit, α-actinin, and Rock2 indicating that their effects on integrin, actin-binding proteins and Rho-kinases are selective and specific. Our findings unveil a selective mechanism by which the PCAIs inhibit the formation of focal adhesions to abrogate cell migration and metastasis.

## RESULTS

### PCAIs decrease the levels of integrin α4 subunit but not integrin β5

We previously reported that PCAIs decrease the levels of the Rho GTPases (RhoA, Cdc42 and Rac1) in H1299 resulting in the suppression of cell invasion and migration [18]. Since RhoA regulates the formation of focal adhesions which are important for migration, invasion and the colonization of new sites in metastasis, we hypothesized that the PCAIs disrupt the levels and activities of key proteins involved in the formation of focal adhesions.

**Figure 1:**
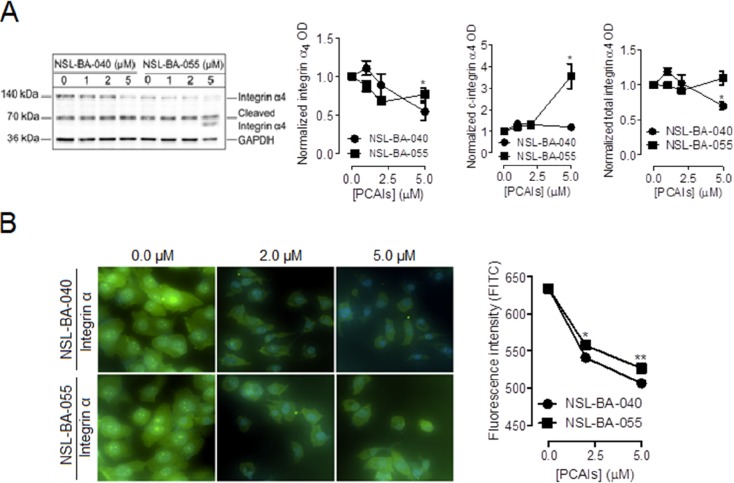
PCAIs diminish the levels of integrin α4 Adherent H1299 cells were exposed to varying concentrations (0-5 μM) of PCAIs for 24 h. Adherent cells were **(A)** lysed in RIPA buffer, protein concentration was determined, lysates containing equal amounts of proteins for each treatment group was subjected to SDS-gel electrophoresis and proteins transferred onto PVDF membranes. Membranes were immuno-probed with an antibody against integrin α4 and the levels of this protein were visualized using HRP-conjugated rabbit IgG secondary antibodies and ECL reagent in Western blotting or **(B)** fixed onto glass slides with a 4 % formaldehyde solution, permeabilized with a 0.3% TritionX-100 solution and then incubated in 1% BSA/0.3% TritionX-100 solution containing an antibody against integrin α4. Cells were then visualized using rabbit IgG Alexa Fluor 488 conjugate and a Nikon *Ti* Eclipse microscope at 40X magnification. The levels of full length integrin α4 (140 kDa), cleaved integrin α4 (70 kDa and 66 kDa) and total integrin α4 (140 kDa, 70 kDa, 66 kDa) were quantified for each treatment group using ImageLab Software and normalized against GAPDH in (A). The NIS Element software was used for quantification in (B) for N = 100 cells in each treatment group. Normalized data was used to generate the graphs shown in GraphPad Prism 5.0. Statistical significance (^*^ p < 0.05; ^**^ p < 0.01) was determined by comparing the mean of each treatment group to untreated control using 1-way ANOVA and post-hoc Dunnett's test.

To begin to understand the role of PCAIs in focal adhesion formation, we examined the effect of NSL-BA-040 and NSL-BA-055 on the levels of integrin α4 and β5 subunits known to be expressed on non-small cell lung cancer cell lines and tissues [19, 20]. In Western blotting assays, we observed that exposure to NSL-BA-055 decreased the levels of full-length α4 by 24%, but increased the levels of the cleaved forms of integrin α4 by 250% (Figure 1A). NSL-BA-040 decreased the levels full-length α4 by 46% but did not alter the levels of the cleaved forms of integrin α4. A quantification of the total levels of integrin α4 protein (full length plus cleaved forms) indicated that exposure to 5 μM NSL-BA-040 resulted in a 30% decrease in the total levels of integrin α4 whereas 5 μM NSL-BA-055 did not alter the total levels of integrin α4 but rather promoted the accumulation of the cleaved forms of this protein.

Although no functional differences in terms of their adhesive properties have yet been reported, the signaling regulated by full-length versus cleaved α integrin subunits have been observed to be different [21, 22]. In immunofluorescent assay, exposure to 5 μM of NSL-BA-040 and NSL-BA-055 resulted in a 21% and 17% decrease, respectively, in fluorescent intensity of integrin α4 when compared to control (Figure 1B). This change in fluorescent intensity was expected for NSL-BA-040 and correlates with the 30% decrease in the overall levels of total integrin α4 (full-length plus cleaved forms) protein observed in Western blotting assays. The decrease in fluorescent intensity induced by NSL-BA-055 was unexpected as in Western blotting, this compound does not alter the total levels of the integrin α4 protein but rather promotes the accumulation of cleaved forms of the protein. Previously, we reported that treatment with 5 μM PCAIs results in the pinching off of vesicles from the plasma membrane containing membrane proteins such as F-actin [18]. The PCAIs may thus induce the pinching-off of vesicles and loss of integrin α4 from membranes which is reflected in immunofluorescent assays (Figure 1B). Western blotting involving the determination of total proteins normalized against GAPDH may not necessarily reflect the potential loss of a small subset of membrane proteins.

**Figure 2:**
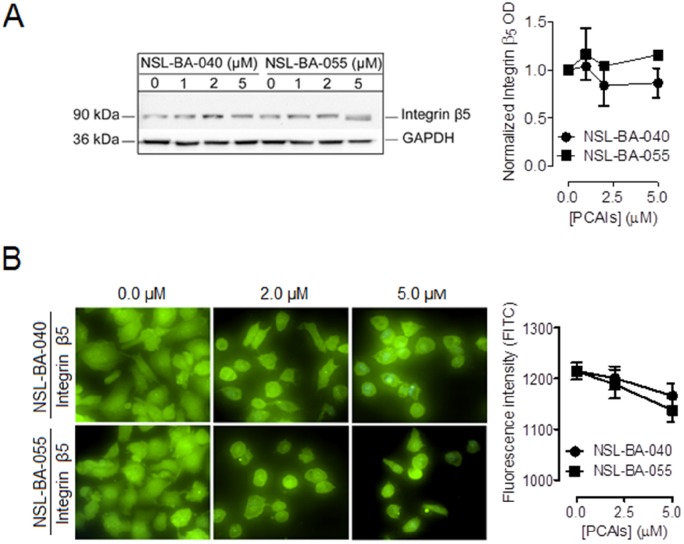
PCAIs do not significantly alter the levels of integrin β5 Adherent H1299 cells were exposed to varying concentrations (0-5 μM) of PCAIs for 24 h. Adherent cells were **(A)** lysed in RIPA buffer, lysate volumes containing equal amounts of proteins were subjected to SDS-gel electrophoresis and proteins transferred onto PVDF membranes. Membranes were incubated in 5% non-fat dry milk solution containing an antibody against integrin β5 and the levels of this protein were visualized using HRP-conjugated rabbit IgG secondary antibodies and ECL reagent in western blotting or **(B)** fixed onto glass slides with a 4 % formaldehyde solution, permeabilized with a 0.3% Trition-X-100 solution and then incubated in a 1% BSA/0.3% TritionX-100 solution containing an antibody against integrin β5. Cells were then visualized using rabbit IgG Alexa Fluor 488 conjugate and a Nikon *Ti* Eclipse microscope at 40X magnification. Protein levels were quantified using ImageLab Software in (A), NIS Element software in (B) for N = 100 cells for each treatment group and then plotted using GraphPad Prism 5.0. Statistical analysis was determined by comparing the mean of untreated control to the mean of all treatment groups using 1-way ANOVA with post-hoc Dunnett's test.

The PCAIs did not significantly alter the levels of the integrin β5 subunit in both Western blotting (Figure 2A) and immunofluorescent assays (Figure 2B) indicating that the PCAIs selectively alter the levels of the α4 integrin subunit but not the β5 integrin subunit.

### PCAIs decrease the levels of actin-binding protein vinculin but not α-actinin

**Figure 3:**
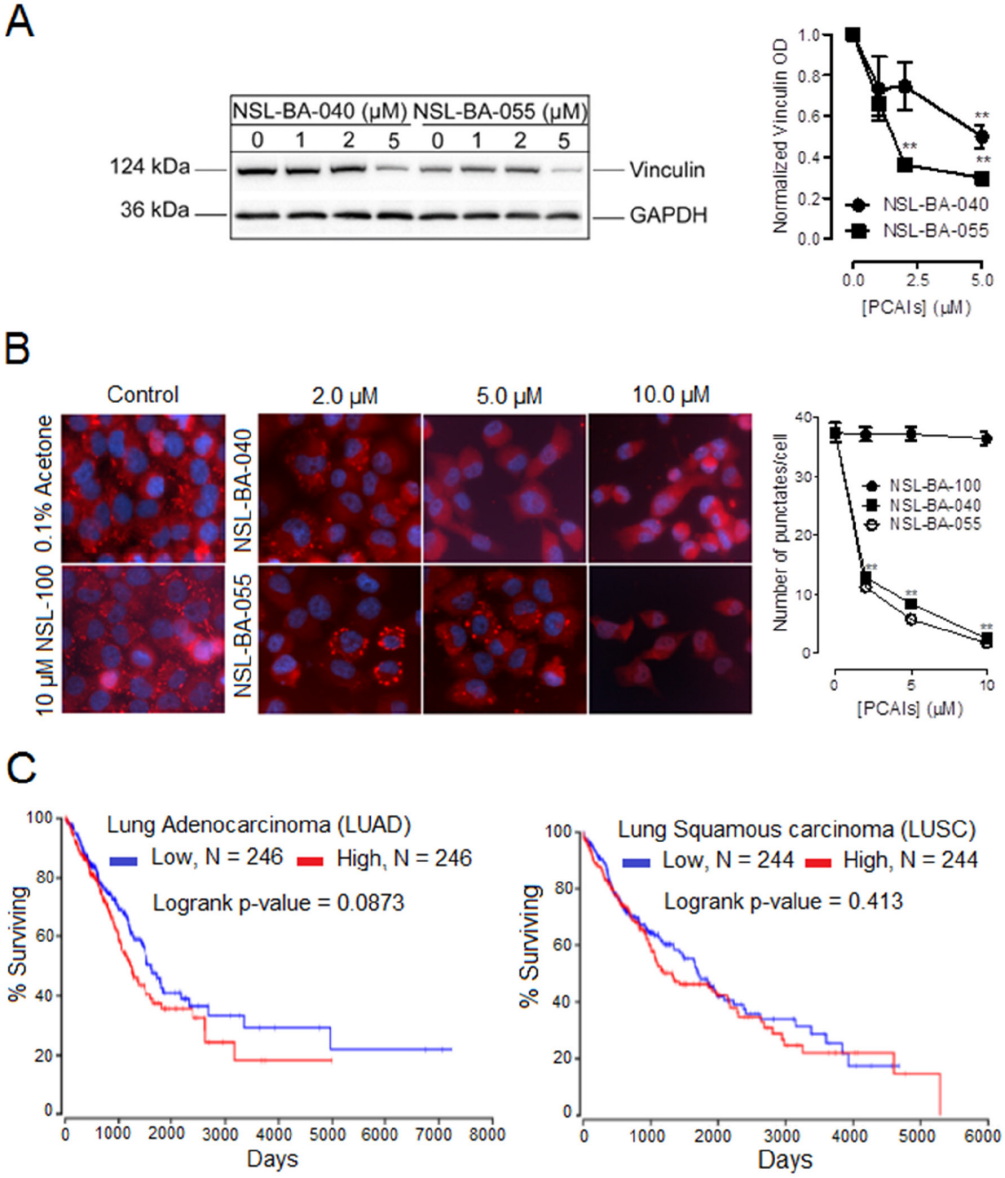
PCAIs diminish the levels of vinculin to suppress the formation of vinculin punctuates at focal adhesion and clinical relevance of vinculin mRNA levels in lung cancer Adherent H1299 cells were exposed to varying concentrations (0-10 μM) of PCAIs for 24 h. Adherent cells were **(A)** lysed in RIPA buffer, lysates containing equal protein amounts were subjected to gel electrophoresis and proteins transferred onto PVDF membranes. PVDF membranes were then incubated with an antibody against vinculin. The levels of vinculin in the lysates were visualized using HRP-conjugated rabbit IgG secondary antibodies and ECL reagent in western blotting or **(B)** fixed onto glass coverslips using a 4 % formaldehyde solution, permeabilized with a 0.3% TX-100 solution and then incubated with an antibody against vinculin in a 1% BSA/0.3% TX-100 solution. Cells were then visualized using rabbit IgG Alexa Fluor 555 conjugate and a Nikon *Ti* Eclipse microscope at 40X magnification. Protein levels for 4 independent experiments in (A) were quantified using ImageLab Software and normalized against GAPDH. In (B), N = 300 cells were analyzed using NIS Element software. Also, in (B), the effects of a control compound (NSL-100), which lacks the farnesyl moiety present in PCAIs, were also determined. Graphs were plotted using GraphPad Prism 5.0. Statistical significance (^**^ p < 0.01) was established by comparing the mean of untreated control to the means of the different treatment groups by 1-way ANOVA with post-hoc Dunnett's test. **(C)** Kaplan plots depicting the relationship between lung cancer survival (LUAD and LUSC) and vinculin mRNA levels were generated from TCGA data from OncoLnc (http://www.oncolnc.org).

Next, we investigated the effects of PCAIs on two actin-binding proteins; vinculin and α-actinin that bridge integrins to the actin cytoskeleton. Whereas α-actinin directly binds integrin and the F-actin cytoskeleton, vinculin does not bind integrin directly but binds onto α-actinin or other adapter proteins to cross-link integrins to the F-actin cytoskeleton. In Western blotting and immunofluorescence assays, we observed a decrease in the levels of vinculin protein and the number of punctuates at focal adhesion sites, respectively. The levels of vinculin protein decreased by 50% and 70% with exposure to 5 μM of NSL-BA-040 and NSL-BA-055, respectively (Figure 3A). The number of vinculin punctates present at focal adhesion points dropped by 76% and 86% after exposure to 5 μM NSL-BA-040 and NSL-BA-055, respectively, when compared to the untreated control (Figure 3B). Bright field images of cells exposed to 5 μM of PCAIs indicate that the bulk of cells remained attached yet rounded (Supplementary Figure 2). Exposure to 10 μM of PCAIs resulted in significant cell detachment, yet some of the cells remained attached. An examination of these adherent cells indicated that 10 μM PCAIs completely inhibited the formation of vinculin punctates, indicating disruption and/or inhibition of the formation of focal adhesions (Figure 3B). Furthermore, we consistently observed sharp demarcations of punctates in untreated controls and cells exposed to 2 μM PCAIs, while treatment with 5 μM and 10 μM of PCAIs resulted in diffused vinculin staining probably due to PCAIs-induced disruption of punctates and dispersion of vinculin into the cytoplasm.

To determine if the farnesyl moiety was required and necessary for the PCAIs-mediated disruption of focal adhesion punctates, we examined the effects of NSL-100 that lacks the farnesyl moiety on vinculin punctates (Figure 3B). As expected, exposure to up to 10 μM of NSL-100 did not significantly change the number of vinculin punctates observed per cell when compared to the untreated control cells.

**Figure 4:**
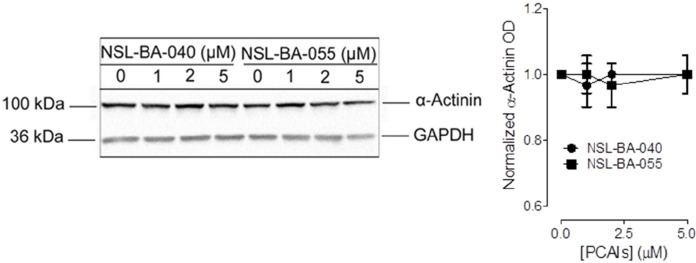
PCAIs do not significantly alter the levels of α-actinin H1299 cells (7 × 10^5^ cells) were plated in complete media into 100 mm cell culture plates and incubated (37°C; 5% CO_2_) overnight. The next day, adherent cells were exposed to varying concentrations (0-5 μM) of PCAIs in experimental media for 24 h. Cells were then washed with a 1x PBS solution and lysed in RIPA buffer supplemented with protein inhibitors. Lysates containing equal amounts of protein were subjected to SDS-gel electrophoresis, proteins transferred onto PVDF membranes. Membranes were blocked in 5% non-fat dry milk for 1 h and incubated with a primary antibody against α-Actinin overnight. The levels of α-Actinin protein under the different conditions were visualized using HRP-conjugated rabbit IgG secondary antibodies and ECL reagent. Protein levels were quantified using ImageLab Software, normalized against GAPDH and then plotted using GraphPad Prism 5.0.

To determine if the PCAIs also alter the levels of other actin-binding proteins, we next examined their effects on α-actinin. In Western blotting assays, the levels of α-actinin in lysates generated from H1299 cells that were exposed to 5 μM of PCAIs were not significantly different from the levels of the protein observed in the untreated control lysates (Figure 4).

Together, these findings indicate that the farnesyl moiety in PCAIs is required and important for the selective decrease in the levels of the actin-binding protein, vinculin.

To assess the clinical relevance of these findings, we wondered if vinculin mRNA or protein levels in patient samples correlate with lung cancer survival. Whereas information on vinculin protein levels and lung cancer survival is still lacking, information on vinculin mRNA levels and lung cancer survival is available from TCGA database. We therefore generated Kaplan-Meier plots from TGCA data (Figure 3C) and observed that there was no significant difference in the survival of patients with lung adenocarcinoma or lung squamous carcinoma exhibiting relatively low or high levels of vinculin mRNA.

The Kaplan plots suggest various possibilities with respect to the role that vinculin may play in cancer progression. One possibility is that its levels may not be important for disease progression. On the other hand, the mRNA levels may not correlate with the actual protein levels, or that the lower levels used for comparison with the higher levels may have already met plateau levels for promoting tumor progression in which case its relevance to progression would not be discernable.

### NSL-BA-055 decreases the levels of Rock1 and its substrate p-MLC-2, but not Rock2, to disrupt focal adhesion formation

The Rock kinases (Rock1 and Rock2) are effectors of RhoA and key signaling regulators of focal adhesion. Rock kinases are activated by RhoA. Upon activation, they phosphorylate MLC-2 to promote actomyosin contractibility and the assembly of integrin-mediated focal adhesion. Based on our previous observation that the PCAIs diminish the levels of RhoA [18], we wondered if the PCAIs may affect the levels of the RhoA effectors, Rock1 and Rock2 and their substrate, MLC-2.

**Figure 5:**
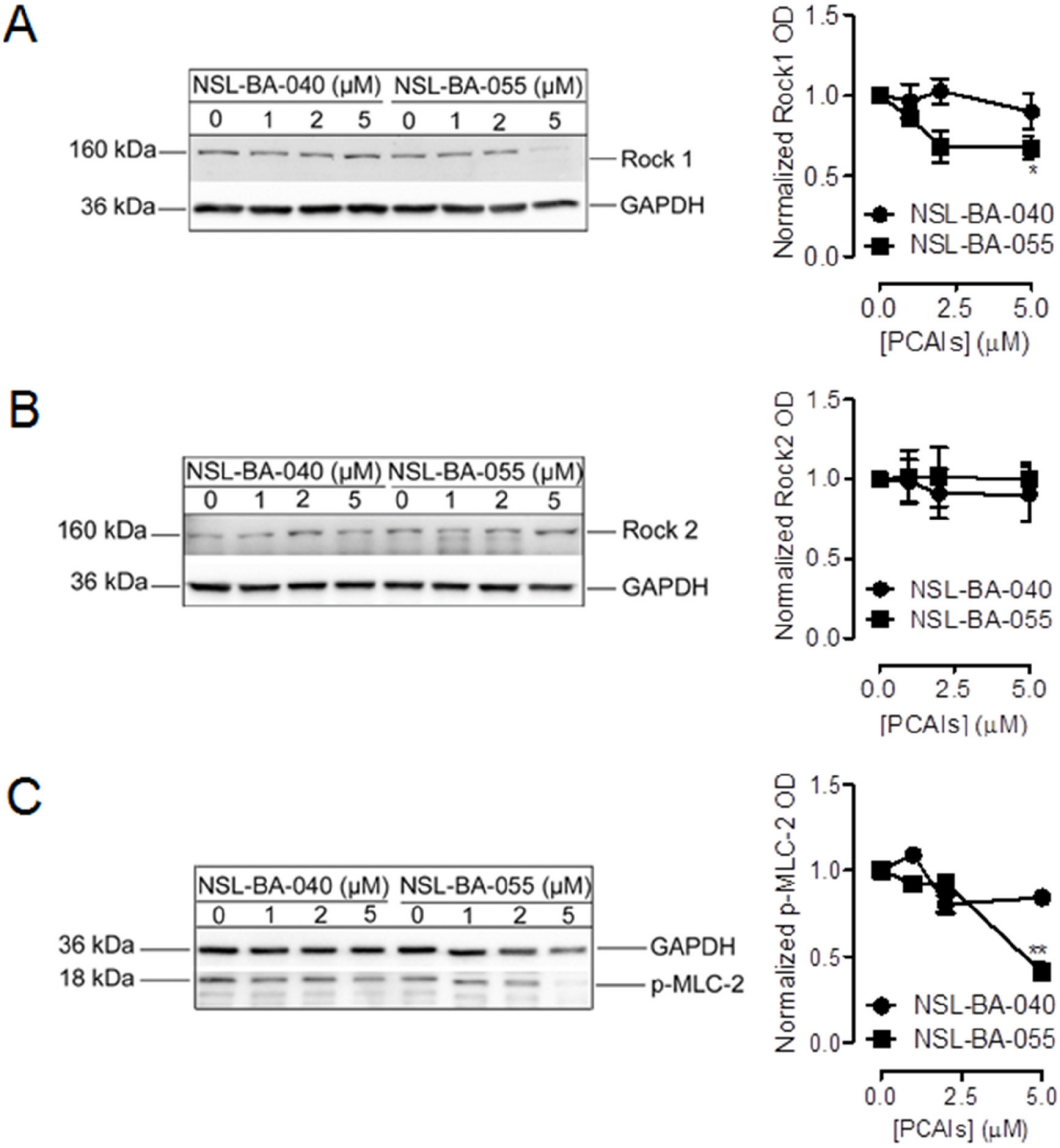
NSL-BA-055 diminishes the levels of the Rock1 and its substrate MLC-2 but not Rock2 H1299 cells (7×10^5^cells/dish) were plated into 100 mm cell culture dishes in complete RPMI media. The cells were incubated (37°C; 5% CO_2_) for 24 h before they were treated with 0.1% acetone (vehicle) or PCAIs (0 – 5 μM) in experimental media for 24 h. Cell lysates were generated by lysis with RIPA buffer supplemented with a protease and phosphatase inhibitor cocktail. Lysates containing equal amounts of proteins were then subjected to SDS-gel electrophoresis, proteins transferred onto PVDF membranes and membranes probed with primary antibodies against **(A)** Rock1, **(B)** Rock2 and **(C)** p-MLC-2. Bound antibodies on immunoblots were visualized using a HRP-Rabbit IgG secondary and ECL-reagents. Protein levels were quantified using ImageLab Software, normalized against GAPDH and then plotted using GraphPad Prism 5.0.

To investigate this possibility, we examined the levels of the Rock1, Rock2 and p-MLC in Western blotting analysis of cell lysates after cell exposure to PCAIs. Interestingly, we observed that exposure to NSL-BA-055 but not NSL-BA-040 resulted in a 25% decrease in the levels of Rock1 kinase (Figure 5A). PCAIs exposure did not alter the levels of the Rock2 proteins (Figure 5B). The PCAI-induced 25% decrease in the levels of Rock1 kinase was accompanied by a 75% concomitant decrease in the level of p-MLC-2 (Figure 5C).

These findings suggest functional differences between the Rock kinases and also indicate a selective role of NSL-BA-055 in suppressing the levels of Rock1 but not Rock2 to inhibit focal adhesion assembly or to enhance its disassembly.

### PCAIs decrease the levels of fascin protein to disrupt filopodia formation and cell migration

In a previous report, we demonstrated that PCAIs diminished filopodia density on cells and blocked cell invasion and migration [18]. Filopodia are finger-like structures at the cell surface formed by tightly bundled parallel actin filaments. The formation of actin filament bundles in filopodia is mediated by the actin-crosslinking protein fascin [23].

**Figure 6:**
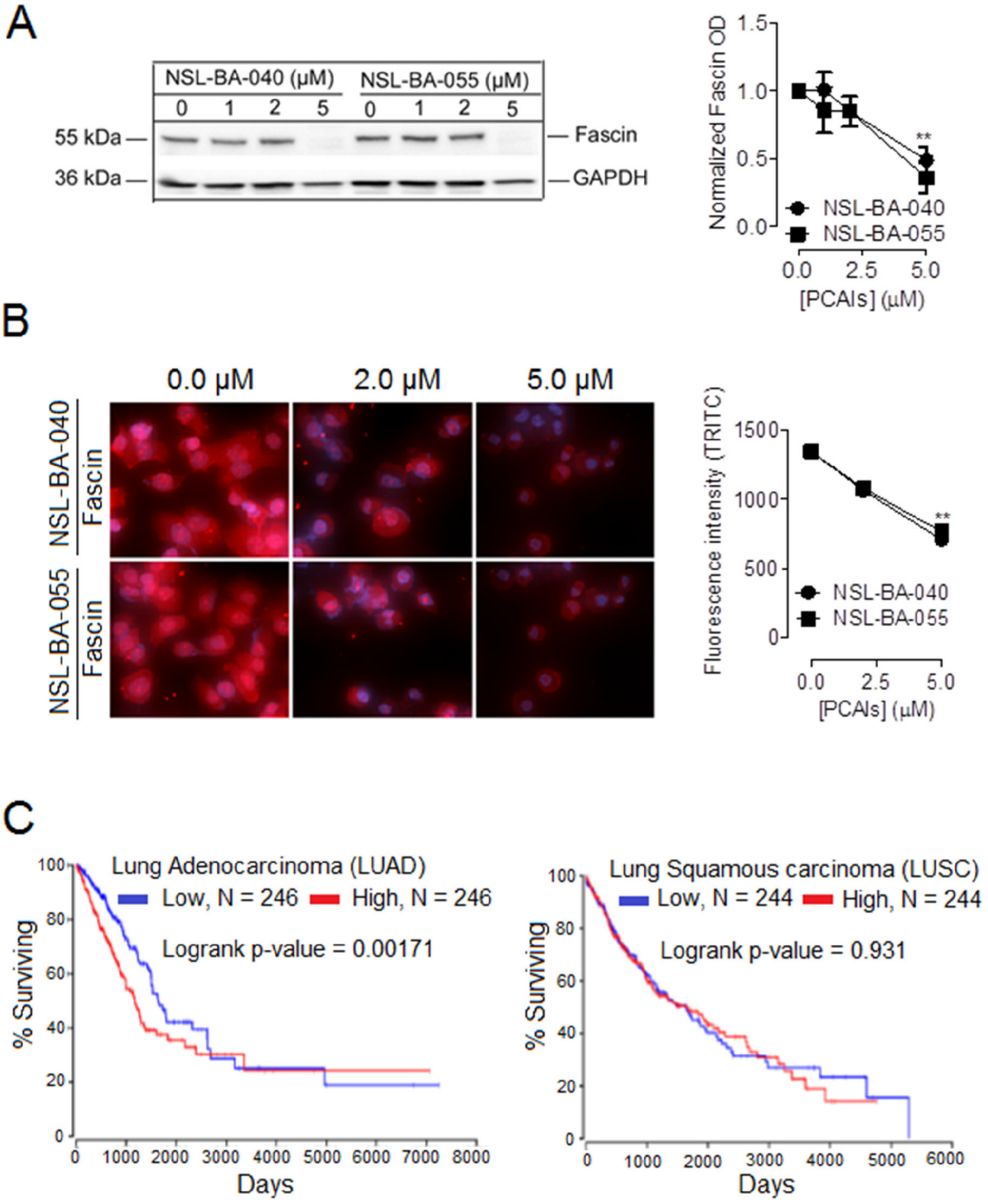
PCAIs-mediated suppression of the levels of fascin protein and clinical relevance of fascin mRNA levels in lung cancer **(A)** H1299 cells at a density of 7×10^5^ cells/dish were plated into 100 mm dishes and incubated (37 °C; 5% CO_2_) overnight. Cells were then treated with either 0.1% acetone or PCAIs (0- 5 μM) for 24 h, then lysed in RIPA buffer supplemented with a protease inhibitor cocktail. Lysates containing equal amounts of protein were subjected to SDS-gel electrophoresis. Proteins transferred onto PVDF membranes where then immunoblotted for fascin and GAPDH (loading control). Band intensities were quantified using ImageLab Software and normalized against GAPDH. **(B)** H1229 cells were plated into the wells of 6-channel ibidi μ-Slides at a density of 1.2 × 10^4^ cells/well. Cells were incubated (37 °C; 5 % CO_2_) overnight to allow them to adhere to slides. The next day, cells were fixed with a 4% Formaldehyde solution, permeabilized using a 0.3% TX-100 solution. Non-specific sites were blocked by incubating the cells with 5% BSA solution for 1h. This was followed by an overnight incubation (4°C with shaking) with a fascin antibody in a 1% BSA/0.3% TritionX-100 solution. Fixed cells were then washed three times with 1x PBS and then incubated with anti-mouse IgG Alexa Fluor 555 conjugate. Fluorescent cells were visualized and captured using a Nikon Ti Eclipse microscope. Fluorescent intensity/cell were quantified using NIS Element software for N = 100 cells under each treatment condition. Graphs and statistical analysis (^**^ p < 0.01) were generated in GraphPad Prism 5.0. **(C)** Kaplan plots depicting the relationship between lung cancer survival (LUAD and LUSC) and fascin mRNA levels were generated from TCGA data from OncoLnc (http://www.oncolnc.org).

To gain more insight on the mechanism by which exposure to PCAIs results in a decrease in the density of filopodia, we wondered whether PCAIs may alter the levels of the fascin protein similarly to vinculin in focal adhesions resulting in the disruption of fascin-mediated cross-linking of actin bundles in filopodia. We investigated this possibility using Western blotting and immunofluorescent assays. In Western blotting assays, we observed a 51% drop in the levels of fascin protein with exposure to NSL-BA-040 and a 64% drop in the levels of the protein with exposure to NSL-BA-055 (Figure 6A). In immunofluorescent assays, we observed a 45% and 47% decrease in fascin fluorescence intensity after 24h exposure to NSL-BA-040 and NSL-BA-055, respectively (Figure 6B).

These findings support the role of PCAIs in disrupting the levels of fascin protein to inhibit F-actin organization, to suppress filopodia and focal adhesions, thus inhibiting cell invasion. Additionally, findings are in agreement with reports that filopodia formation and integrin-mediated focal adhesions cooperatively regulate cell motility and invasion [24].

Our findings that PCAIs exposure suppresses the levels of fascin protein, coupled with reports that fascin protein is overexpressed in various cancer types [25–31] raises the possibility that the levels of fascin mRNA correlate with poor survival in lung cancer patients. We thus generated Kaplan plots from TCGA data (Figure 6C). These plots reveal that the levels of fascin mRNA significantly correlate with survival of patients with lung adenocarcinoma (LUAD) but not for patients with lung squamous carcinoma (LUSC). The Kaplan plots suggest that the upregulation of fascin protein in NSCLC is both transcriptionally-and/or translationally-regulated.

## DISCUSSION

Tumor metastasis plays a major role in morbidity and deaths from cancer. The process of metastasis involves cancer cell migration and invasion of surrounding tissues.

Integrins, a group of cell surface receptors, mediate the communication between the ECM and the cell interior. Integrins, by connecting the ECM to the actin cytoskeleton, mediate cell migration, invasion, angiogenesis and adhesion. Changes in integrin expression are associated with tumor growth and metastasis in various types of cancers [9, 14, 15]. Tumor progression is related, therefore, to structural and biochemical dysregulation of integrins. Therapeutic agents that affect integrin cell expression and function may be of interest and are being explored as inhibitors of tumor growth, metastasis and angiogenesis [32, 33].

We demonstrated earlier that a novel class of synthetic small molecules, known as the PCAIs, diminish the levels of the Rho proteins (Rac1, Cdc42 and RhoA) and suppress F-actin remodeling in NSCLC cells thereby blocking cell migration and invasion [18]. PCAIs disrupt F-actin organization in lamellipodia and filopodia structures to abrogate cell migration.

In this report, we investigated the effect of PCAIs on focal adhesions. We uncover a specific mechanism by which the PCAIs achieve their role in blocking cell migration by demonstrating that the PCAIs selectively alter

1. the levels of integrin α4 but not β5;
2. the RhoA-Rock1-pMLC-2 signaling pathway;
3. levels of the F-actin cross-linking protein vinculin but not α-actinin;
4. the levels of the fascin protein, an independent prognostic marker for non-small cell lung cancer, to stop the motility and invasiveness of NSCLC cells.

To understand the effects of PCAIs on focal adhesion assembly and disassembly, we examined their effects on integrin subunits α and β. Earlier findings show that the expression and signaling of integrins change in response to environmental factors [12–15, 34–36]. Because cancer cells manipulate the levels and activity of integrins, efforts have been geared towards developing therapeutics agents that modulate integrin structure and functions. Whereas the PCAIs did not significantly alter the levels of β5 integrin, we observed a 24% decrease and 250% increase in the levels of full-length and cleaved forms of α4 integrin, respectively, on exposure of lung cancer cells to NSL-BA-055. NSL-BA-040 elicited a 46% decrease in the levels of the full length integrin α4 protein, however it did not promote the accumulation of the cleaved forms of this protein. The α4 integrin is one of the integrins that undergoes proteolytic cleavage and can be expressed at the cell surface as a 150 kDa or 140 kDa intact form or a 70 kDa or 80 kDa C-terminal cleaved form [37]. Additionally, a 34 kDa cleaved form of the integrin α4 protein has been described [38] suggesting that several cleavage sites or forms of cleaved integrin α4 exist. We observed a full length form at 140 kDa and two cleaved forms of the α4 protein at 70 kDa and 66 kDa in Western blotting assays. Exposure to PCAIs significantly diminished the levels of the full-length α4 integrin. Furthermore, exposure to 5 μM NSL-BA-055 resulted in an increase in the levels of the cleaved forms of integrin α4 whereas NSL-BA-040 did not enhance the cleavage of integrin α4. These observations suggest that the PCAIs promote the downregulation, secretion or degradation of full length integrin α4 and NSL-BA-055 enhances the proteolytic cleavage of full-length α4 integrin to 70 kDa and 66 kDa forms. It has been postulated that an intracellular endogenous subtilisin-like protease is responsible for the C-terminal cleavage of the α4 subunit [39]. Therefore, NSL-BA-055 may increase the enzymatic activity of such a protease to promote the accumulation of cleaved forms of integrin α4. Another possibility is that the NSL-BA-055 may disrupt protein complexes involving integrin α4 thereby exposing it to the proteolytic enzyme. Interestingly, no differences in the binding of the full-length and cleaved forms of integrin α4 to ECM substrates have been found [21, 39].

Cleavage of the α4 integrin subunit may alter its interaction with cellular binding partners and thus it's signaling. NSL-BA-055-promoted cleavage of α4 integrin may alter the signaling of this protein with a goal of disrupting the formation and turnover of focal adhesion. In support of this, Berthet et al. [22] observed a difference in the signaling of cleaved versus full-length forms of αv, α3 and α6 integrin subunits. Our findings that the PCAIs diminish the levels of full-length integrin α4, NSL-BA-055 increases the levels of cleaved integrin α4, and that the PCAIs do not affect the levels of β5 is novel and points to its specificity and the ability of the PCAIs effects to discriminate between the different integrin subunits. These findings are significant as we do not know of any reports of agents that discriminate between the α and β integrin subunits. Additionally, these findings predict that the PCAIs alter signaling mediated by integrin α4 by diminishing the levels of the full-length form or promoting its proteolytic cleavage.

Our observations that the PCAIs decrease the levels of full-length and NSL-BA-055 increases the levels of cleaved forms of α4 integrin predict PCAIs-induced changes in signaling and provide a rationale for investigating the RhoA-Rock-pMLC-2 signaling pathway for potential therapeutic applications. RhoA-Rock-pMLC-2 signaling pathway has been reported to modulate focal adhesion assembly and disassembly during cell motility [16]. The Rock kinases which are downstream effectors of RhoA, modulate the contractibility of the actomyosin cytoskeleton and thus assembly and disassembly of focal adhesions during cell migration [16, 40, 41]. We investigated the ability of PCAIs to alter this signaling pathway to disrupt the formation of focal adhesion. Whereas NSL-BA-040 did not alter the RhoA-Rock-pMLC-2 signaling pathway, NSL-BA-055 significantly suppressed the RhoA-Rock1-pMLC-2 signaling pathway as evidenced by a decrease in the levels of phosphorylated myosin light chain-2 (pMLC-2) proteins. These findings, point to differences in the mechanism of action of the PCAIs. Additionally, whereas NSL-BA-055 significantly diminished the levels of Rock 1, it did not alter the levels of Rock 2. The NSL-BA-055 induced decrease in the levels of Rock 1 kinase, translates to a significant decrease in the phosphorylation of its substrate (MLC-2). An explanation for the observed effect of NSL-BA-055 on Rock 1, but not Rock 2, is that the Rock kinases (Rock1 and Rock2) likely play separate roles in different cell types. It is possible that in lung cancer, Rock 1 may be the major regulator of the RhoA-Rock-pMLC-2 signaling pathway in focal adhesion formation. In line with this reasoning, Rock1 overexpression has been reported in 73.3% of non-small cell lung cancer samples [42]. We are currently working on identifying other signaling pathways that are affected by the PCAIs.

To expand our understanding of the role of PCAIs in focal adhesion formation, we investigated the effects of the PCAIs on the actin-binding proteins, vinculin and α-actinin. Actin-binding proteins link integrins at the cell surface to the F-actin cytoskeleton and are important for transmitting mechanical forces and orchestrating signaling events. Changes in the levels of actin-binding proteins may in turn alter the strength and integrity of focal adhesions. The actin-binding protein α-actinin interacts with β integrin cytoplasmic tails, F-actin and vinculin at focal adhesions [43–46]. The binding of α-actinin to integrin on its own is not sufficient to recruit F-actin [46, 47]. The recruitment of vinculin to focal adhesions is thus important and required for focal adhesion formation [48]. Vinculin, one of the most abundant proteins at focal adhesions, interacts with F-actin, α-actinin, talin and other binding partners [44, 49] and mediates the recruitment of core focal adhesion proteins to regulate cell adhesion, migration and polarization [50]. In assays where we examined the effects of PCAIs on F-actin-binding proteins, we observed that the PCAIs did not alter the levels of the α-actinin protein but dramatically diminished the levels of vinculin protein and the number of vinculin punctates observed at focal adhesions. To further elucidate the mechanism of PCAIs-induced loss of vinculin at focal adhesions, we examined the effects of a control compound, NSL-100, which lacks the farnesyl moiety present in PCAIs on vinculin levels and punctates. We observed that NSL-100 did not significantly alter the levels of vinculin protein and the number of vinculin punctates at focal adhesions, indicating that the farnesyl moiety is important and essential for the PCAIs-mediated decrease in vinculin protein levels.

To begin to understand the clinical relevance of alterations in vinculin protein levels in relation to lung cancer survival, we examined Kaplan plots generated from TCGA data. Although there was no correlation between the levels of vinculin mRNA and lung cancer survival, this does not necessarily mean that vinculin levels have no clinical relevance as the levels of mRNA do not always correlate with protein levels. Further studies are therefore needed to further understand its clinical relevance.

Vinculin has been reported to associate with membrane lipids [51–54] and its binding to phospholipids has been postulated to be a potential mechanism for its activation [52]. Upon activation, vinculin is thought to interact with its binding partners and these interactions are important in stabilizing the vinculin protein at focal adhesions [48]. Since vinculin is essential at focal adhesions, providing the mechanical force for traction and strengthening of integrin-F-actin linkages, PCAIs-mediated depletion of vinculin may weaken integrin-F-actin linkages, thereby inhibiting cell motility and invasion. Our observation that the PCAIs do not alter the levels of the α-actinin protein points to their specificity in affecting only some actin-binding proteins. This is in agreement with our finding that the PCAIs do not alter the levels of β5 integrin since α-actinin binds the cytoplasmic tail of β integrins [45, 46]. Direct binding of the β-integrin subunit to α-actinin may stabilize this pair so that they remain unaffected by PCAIs.

Our findings that the PCAIs suppress the levels of the actin-crosslinking protein vinculin in focal adhesions made us wonder if the PCAIs similarly alter the levels of the actin-crosslinking protein fascin that regulates the formation of actin filament bundles in filopodia [55]. Previously, we demonstrated that the PCAIs disrupt the formation of filopodia structures to block cell migration [18]. Integrin-mediated adhesion has been reported to stabilize filopodia structures and to work cooperatively with filopodia to promote cell migration [24]. In experiments examining the effects of PCAIs on the fascin protein, we observed that the PCAIs in a concentration-dependent manner, markedly diminished the levels of fascin. These results are of clinical significance as among the filopodia-regulating proteins, fascin has been strongly implicated in tumor progression and metastasis. Fascin levels have been reported to be upregulated in more aggressive and metastatic cancers and fascin has been described as a significant and independent prognostic indicator of the outcome of non-small cell lung cancer [23, 56]. The fact that exposure to the PCAIs diminishes the levels of the fascin protein, and our previous findings that the PCAIs suppress the number of filopodia per cell [18] points to a clinical potential of the PCAIs in the treatment of metastatic non-small cell lung cancer. Additionally, Kaplan plots generated from TCGA data indicate that the levels of fascin mRNA inversely correlate with the survival of patients with lung adenocarcinoma (LUAD) but not lung squamous carcinoma (LUSC). This observation together with the established clinical relevance of fascin [23, 56] and our findings that the PCAIs diminish the levels of fascin protein in the H1299 NSCLC in a concentration-dependent manner, suggest that the PCAIs may alter the levels of fascin mRNA and/or the stability of this protein to suppress its function in filopodia formation.

In conclusion, we elucidate a comprehensive mechanism by which the PCAIs disrupt cell migration and invasion. NSL-BA-055, by diminishing the levels of full-length integrin α4, enhancing the proteolytic cleavage of integrin α4, suppressing the Rho-Rock1-pMLC-2 signaling pathway, and the levels of vinculin and fascin proteins disrupts focal adhesions and filopodia structures, and abrogates cell migration and invasion. NSL-BA-040, by diminishing the levels of full-length integrin α4, suppressing the levels of vinculin and fascin proteins disrupts focal adhesions and filopodia structures to inhibit cell migration and invasion. In our previous work, we showed that the PCAIs induced the pinching-off of F-actin vesicles from cell membranes and thus loss of F-actin resulting in loss of filopodia and lamellipodia [18]. Cumulatively, our data indicate that the PCAIs enhance F-actin loss probably by diminishing the levels of specific F-actin-binding proteins to disrupt lamellipodia, filopodia and focal adhesion structures in NSCLC cells. Identification of the primary molecular target(s) of the PCAIs now becomes relevant in anticancer strategies. This study provides strong mechanistic evidence that the PCAIs alter the levels of a number of proteins involved in integrin signaling, RhoA, Rac1 and Cdc42 signaling, F-actin organization at filopodia, lamellipodia and focal adhesions thereby modulating processes such as cell adhesion, migration and invasion.

## MATERIALS AND METHODS

### Cell culture

NCI-H1299 cells were obtained from American Type Culture Collection (ATCC – Manassas, VA) and cultured in RPMI basal medium supplemented with 10% FBS (complete medium). Cells were maintained at 37°C/5% CO_2_ in a cell culture incubator and treated in RPMI basal medium supplemented with 5% FBS (treatment/experimental medium). Only adherent cells were used in all assays described.

### Western blotting assays

Focal adhesion formation is mediated by several cellular proteins including integrins, vinculin, α-actinin, and the Rock kinases. The levels and activities of integrins [9–15], vinculin [57–63] and the Rock kinases [64–68] have been reported to be dramatically altered in various cancer types to promote tumorigenesis and metastasis. Compounds that affect the levels or activities of some or all of these proteins may have therapeutic benefits in the treatment of cancers. We thus examined the levels of the focal adhesion proteins after exposure to our novel class of compounds, PCAIs [18] (see Supplementary Figure 1 for structures). In these assays, NCI-H1299 cells were plated in complete medium into 100 mm cell culture dishes at a cell density of 7.0 × 10^5^ cells/dish and then incubated for 24 h to allow the cells to adhere. Adherent cells were treated with varying concentrations of PCAIs (0 - 5μM) in experimental medium for 24 h. Cells were washed with PBS and then lysed in RIPA buffer supplemented with protease and phosphatase inhibitors (Thermo Scientific, MA). Cell lysates containing equal protein amounts were boiled in laemmli buffer and then subjected to SDS-PAGE. Proteins were transferred unto Polyvinylidene difluoride (PVDF) membranes and immunoblotted using antibodies against the following proteins: Vinculin (Cell Signaling Technology – E1E9V), Fascin (Cell Signaling Technology – 55k-2), Rock1 (Cell Signaling Technology - C8F70), Rock2 (Cell Signaling Technology - D1B1), alpha-Actinin (D6F6), Integrin alpha 4 (Cell Signaling - D2E1), Integrin beta 5 (Cell Signaling Technology - D24A5), phospho-Myosin Light Chain 2 (Cell Signaling Technology - Ser19) and GAPDH (GeneTex, GTX100118). Bound antibodies were visualized using horseradish peroxidase (HRP)-linked rabbit (Santa Cruz, sc-2004; Cell Signaling Technology) and mouse IgGs (Cell Signaling Technology) and ECL reagents (BioRad, CA) per manufacturer's recommendation. Band intensities were quantified using the ImageLab analysis software (BioRad, CA) and normalized against corresponding band intensities of GAPDH.

### Immunofluorescence assays

To examine the effects of PCAIs on focal adhesion complexes, we captured fluorescent images of NCI-H1299 cells immuno-stained against the focal adhesion receptor proteins integrin α4 and integrin β5, and the focal adhesion F-actin-crosslinking protein, vinculin, after 24 h exposure of cells to PCAIs. Since integrins cooperate with filopodia during cell motility, we also examined the effects of PCAIs on the filopodia F-actin crosslinking protein, fascin. In these assays, NCI-H1299 cells were seeded in complete medium on either glass coverslips placed in the wells of a 24-well plate at a density of 1.0 × 10^4^ cells/well or onto 6-channel i*bidi* μ-Slides at a density of 1.2 × 10^4^ cells/well. Cells were incubated at 37°C/5% CO_2_ for 24 h and then treated with varying concentrations of the PCAIs; NSL-BA-040 or NSL-BA-055 (0-10 μM) in treatment medium for an additional 24 h. In some experiments, cells were also treated with varying concentrations of the control compound, NSL-100 (0-10 μM), which has a similar structure to the PCAIs but lacks the farnesyl moiety present in PCAIs [18]. At the end of every treatment, cells were washed three times with 1x PBS, fixed onto glass coverslips or *ibidi* μ-Slides by incubating them with a 4% formaldehyde solution for 20 mins at room temperature. The cells were then permeabilized by incubating with a 0.3% TritionX-100 solution for 10 mins. Non-specific protein sites were blocked by incubating in a 5% BSA solution for 1 h. After blocking, the cells were incubated overnight with an antibody against Integrin alpha 4 (Cell Signaling Technology - D2E1), Integrin beta 5 (Cell Signaling Technology - D24A5); Vinculin (Cell Signaling Technology – E1E9V); Fascin (Cell Signaling Technology – 55k-2) in 1% BSA/0.3% TritionX-100 solution. After this incubation, the coverslips were washed three times with 1x PBS and then incubated overnight with either anti-rabbit IgG Alexa Fluor 488 conjugate (Cell Signaling Technology), rabbit IgG Alexa Fluor 555 conjugate (Cell Signaling Technology, MA) or anti-mouse IgG Alexa Fluor 555 conjugate (Cell Signaling Technology, MA). Fixed cells were again washed three times with 1x PBS and the coverslips were mounted unto microscope slides using mounting media containing DAPI (Vector Laboratories, H-1200) which stains nuclei blue. Cells on *ibidi* μ-slides were incubated with a 10 μg/mL Hoechst dye solution for 1h, then washed three times with 1x PBS. Slides were visualized using a fluorescent Nikon *Ti* Eclipse microscope. Fluorescent intensity per cell was quantified for 100 or 300 cells under each treatment condition as specified in the figure legends.

### Statistical analyses

All results are the means ± SEM of four independent assays. The means of measured values of each treatment group were compared to their respective vehicle treated controls by One-Way ANOVA with Dunnett's post hoc test. Means were considered to be significantly different from one another when p values were less than 0.05.

### Vinculin/Fascin mRNA expression and lung cancer analysis using TCGA data

To explore survival correlation of lung cancer with mRNA levels of vinculin and fascin genes, we generated Kaplan-Meier plots using OncoLnc (http://www.oncolnc.org). To generate these plots, the lower and upper mRNA expression were both set at 50 percentile. OncoLnc is a newly available resource for linking The Cancer Genomic Atlas (TCGA) survival data to mRNA expression [69].

## SUPPLEMENTARY MATERIALS FIGURES



## References

[1] Yilmaz M, Christofori G, Lehembre F (2007). Distinct mechanisms of tumor invasion and metastasis. Trends Mol Med.

[2] Wu C (2007). Focal adhesion: a focal point in current cell biology and molecular medicine. Cell Adhesion Mig.

[3] Huttenlocher A, Horwitz AR (2011). Integrins in cell migration. Cold Spring Harbor Perspect Biol.

[4] Bosman FT (1993). Integrins: cell adhesives and modulators of cell function. Histochem J.

[5] Elliott T, Sethi T (2002). Integrins and extracellular matrix: a novel mechanism of multidrug resistance. Exp Rev Anticancer The.

[6] Dalton SL, Scharf E, Briesewitz R, Marcantonio EE, Assoian RK (1995). Cell adhesion to extracellular matrix regulates the life cycle of integrins. Mol Biol Cell.

[7] Hynes RO (2002). Integrins: bidirectional, allosteric signaling machines. Cell.

[8] Goldmann WH, Auernheimer V, Thievessen I, Fabry B (2013). Vinculin, cell mechanics and tumour cell invasion. Cell Biol Int.

[9] Guo W, Giancotti FG (2004). Integrin signalling during tumour progression. Nat Rev Mol Cell Biol.

[10] Janes SM, Watt FM (2006). New roles for integrins in squamous-cell carcinoma. Nat Rev Cancer.

[11] Caswell P, Norman J (2008). Endocytic transport of integrins during cell migration and invasion. Trends Cell Biol.

[12] Brooks PC, Clark RA, Cheresh DA (1994). Requirement of vascular integrin alpha v beta 3 for angiogenesis. Science.

[13] Serini G, Trusolino L, Saggiorato E, Cremona O, De Rossi M, Angeli A, Orlandi F, Marchisio PC (1996). Changes in integrin and E-cadherin expression in neoplastic versus normal thyroid tissue. J Nat Cancer Inst.

[14] Tennenbaum T, Weiner AK, Belanger AJ, Glick AB, Hennings H, Yuspa SH (1993). The suprabasal expression of alpha 6 beta 4 integrin is associated with a high risk for malignant progression in mouse skin carcinogenesis. Cancer Res.

[15] Mizejewski GJ (1999). Role of integrins in cancer: survey of expression patterns. Proc Soc Exp Biol Med.

[16] Chrzanowska-Wodnicka M, Burridge K (1996). Rho-stimulated contractility drives the formation of stress fibers and focal adhesions. J Cell Biol.

[17] American Cancer Society (2017). Non-Small Cell Lung Cancer.

[18] Ntantie E, Fletcher J, Amissah F, Salako OO, Nkembo AT, Poku RA, Ikpatt FO, Lamango NS (2017). Polyisoprenylated cysteinyl amide inhibitors disrupt actin cytoskeleton organization, induce cell rounding and block migration of non-small cell lung cancer. Oncotarget.

[19] Guo L, Zhang F, Cai Y, Liu T (2009). Expression profiling of integrins in lung cancer cells. Pathol Res Prac.

[20] Falcioni R, Cimino L, Gentileschi MP, D'Agnano I, Zupi G, Kennel SJ, Sacchi A (1994). Expression of beta 1, beta 3, beta 4, and beta 5 integrins by human lung carcinoma cells of different histotypes. Exp Cell Res.

[21] Parker CM, Pujades C, Brenner MB, Hemler ME (1993). Alpha 4/180, a novel form of the integrin alpha 4 subunit. The J Biol Chem.

[22] Berthet V, Rigot V, Champion S, Secchi J, Fouchier F, Marvaldi J, Luis J (2000). Role of endoproteolytic processing in the adhesive and signaling functions of alphavbeta5 integrin. J Biol Chem.

[23] Machesky LM, Li A (2010). Fascin: Invasive filopodia promoting metastasis. Comm Integ Biol.

[24] Arjonen A, Kaukonen R, Ivaska J (2011). Filopodia and adhesion in cancer cell motility. Cell Adhes Mig.

[25] Iguchi T, Aishima S, Taketomi A, Nishihara Y, Fujita N, Sanefuji K, Sugimachi K, Yamashita Y, Maehara Y, Tsuneyoshi M (2009). Fascin overexpression is involved in carcinogenesis and prognosis of human intrahepatic cholangiocarcinoma: immunohistochemical and molecular analysis. Hum Pathol.

[26] Iguchi T, Aishima S, Umeda K, Sanefuji K, Fujita N, Sugimachi K, Gion T, Taketomi A, Maehara Y, Tsuneyoshi M (2009). Fascin expression in progression and prognosis of hepatocellular carcinoma. J Surg Oncol.

[27] Tsai WC, Chao YC, Sheu LF, Chang JL, Nieh S, Jin JS (2007). Overexpression of fascin-1 in advanced colorectal adenocarcinoma: tissue microarray analysis of immunostaining scores with clinicopathological parameters. Dis Mark.

[28] Chen SF, Yang SF, Li JW, Nieh PC, Lin SY, Fu E, Bai CY, Jin JS, Lin CY, Nieh S (2007). Expression of fascin in oral and oropharyngeal squamous cell carcinomas has prognostic significance - a tissue microarray study of 129 cases. Histopathology.

[29] Daponte A, Kostopoulou E, Papandreou CN, Daliani DD, Minas M, Koukoulis G, Messinis IE (2008). Prognostic significance of fascin expression in advanced poorly differentiated serous ovarian cancer. Anticancer Res.

[30] Gunal A, Onguru O, Safali M, Beyzadeoglu M (2008). Fascin expression [corrected] in glial tumors and its prognostic significance in glioblastomas. Neuropathology.

[31] Zou J, Yang H, Chen F, Zhao H, Lin P, Zhang J, Ye H, Wang L, Liu S (2010). Prognostic significance of fascin-1 and E-cadherin expression in laryngeal squamous cell carcinoma. Eur J Cancer Prev.

[32] Millard M, Odde S, Neamati N (2011). Integrin targeted therapeutics. Theranostics.

[33] Sawada K, Ohyagi-Hara C, Kimura T, Morishige K (2012). Integrin inhibitors as a therapeutic agent for ovarian cancer. J Oncol.

[34] Adachi M, Taki T, Huang C, Higashiyama M, Doi O, Tsuji T, Miyake M (1998). Reduced integrin alpha3 expression as a factor of poor prognosis of patients with adenocarcinoma of the lung. J Clin Oncol.

[35] Tei C, Maruyama T, Kuji N, Miyazaki T, Mikami M, Yoshimura Y (2003). Reduced expression of alphavbeta3 integrin in the endometrium of unexplained infertility patients with recurrent IVF-ET failures: improvement by danazol treatment. J Assist Repro Genet.

[36] Fang Z, Yao W, Fu Y, Wang LY, Li Z, Yang Y, Shi Y, Qiu S, Fan J, Zha X (2010). Increased integrin alpha5beta1 heterodimer formation and reduced c-Jun expression are involved in integrin beta1 overexpression-mediated cell growth arrest. J Cell Biochem.

[37] Pujades C, Teixido J, Bazzoni G, Hemler ME (1996). Integrin alpha 4 cysteines 278 and 717 modulate VLA-4 ligand binding and also contribute to alpha(4/180) formation. Biochem J.

[38] Altevogt P, Hubbe M, Ruppert M, Lohr J, von Hoegen P, Sammar M, Andrew DP, McEvoy L, Humphries MJ, Butcher EC (1995). The alpha 4 integrin chain is a ligand for alpha 4 beta 7 and alpha 4 beta 1. J Exp Med.

[39] Teixido J, Parker CM, Kassner PD, Hemler ME (1992). Functional and structural analysis of VLA-4 integrin alpha 4 subunit cleavage. J Biol Chem.

[40] Zhong C, Kinch MS, Burridge K (1997). Rho-stimulated contractility contributes to the fibroblastic phenotype of Ras-transformed epithelial cells. Mol Biol Cell.

[41] Totsukawa G, Yamakita Y, Yamashiro S, Hartshorne DJ, Sasaki Y, Matsumura F (2000). Distinct roles of ROCK (Rho-kinase) and MLCK in spatial regulation of MLC phosphorylation for assembly of stress fibers, focal adhesions in 3T3 fibroblasts. J Cell Biol.

[42] Yan SG, Meng H, Cheng L, Zhe L, Cao G, Yan W, Xin H (2017). Role of Rock 1 protein in non-small cell lung cancer. Biomed Res.

[43] Burridge K, Chrzanowska-Wodnicka M (1996). Focal adhesions, contractility, and signaling. Ann Rev Cell Dev Biol.

[44] Jockusch BM, Bubeck P, Giehl K, Kroemker M, Moschner J, Rothkegel M, Rudiger M, Schluter K, Stanke G, Winkler J (1995). The molecular architecture of focal adhesions. Ann Rev Cell Dev Biol.

[45] Pavalko FM, Chen NX, Turner CH, Burr DB, Atkinson S, Hsieh YF, Qiu J, Duncan RL (1998). Fluid shear-induced mechanical signaling in MC3T3-E1 osteoblasts requires cytoskeleton-integrin interactions. Am J Physiol.

[46] Cattelino A, Albertinazzi C, Bossi M, Critchley DR, de Curtis I (1999). A cell-free system to study regulation of focal adhesions and of the connected actin cytoskeleton. Mol Biol Cell.

[47] Lewis JM, Schwartz MA (1995). Mapping in vivo associations of cytoplasmic proteins with integrin beta 1 cytoplasmic domain mutants. Mo Biol Cell.

[48] Carisey A, Ballestrem C (2011). Vinculin, an adapter protein in control of cell adhesion signalling. Eur J Cell Biol.

[49] Bubeck P, Pistor S, Wehland J, Jockusch BM (1997). Ligand recruitment by vinculin domains in transfected cells. J Cell Sci.

[50] Carisey A, Tsang R, Greiner AM, Nijenhuis N, Heath N, Nazgiewicz A, Kemkemer R, Derby B, Spatz J, Ballestrem C (2013). Vinculin regulates the recruitment and release of core focal adhesion proteins in a force-dependent manner. Curr Biol.

[51] Tempel M, Goldmann WH, Isenberg G, Sackmann E (1995). Interaction of the 47-kDa talin fragment and the 32-kDa vinculin fragment with acidic phospholipids: a computer analysis. Biophys J.

[52] Johnson RP, Niggli V, Durrer P, Craig SW (1998). A conserved motif in the tail domain of vinculin mediates association with and insertion into acidic phospholipid bilayers. Biochem.

[53] Diez G, List F, Smith J, Ziegler WH, Goldmann WH (2008). Direct evidence of vinculin tail-lipid membrane interaction in beta-sheet conformation. Biochem Biophys Res Comm.

[54] Diez G, Kollmannsberger P, Mierke CT, Koch TM, Vali H, Fabry B, Goldmann WH (2009). Anchorage of vinculin to lipid membranes influences cell mechanical properties. Biophys J.

[55] Vignjevic D, Kojima S, Aratyn Y, Danciu O, Svitkina T, Borisy GG (2006). Role of fascin in filopodial protrusion. J Cell Biol.

[56] Pelosi G, Pastorino U, Pasini F, Maissoneuve P, Fraggetta F, Iannucci A, Sonzogni A, De Manzoni G, Terzi A, Durante E, Bresaola E, Pezzella F, Viale G (2003). Independent prognostic value of fascin immunoreactivity in stage I nonsmall cell lung cancer. Br J Cancer.

[57] Critchley DR (2004). Cytoskeletal proteins talin and vinculin in integrin-mediated adhesion. Biochem Soc Trans.

[58] Coll JL, Ben-Ze'ev A, Ezzell RM, Rodriguez Fernandez JL, Baribault H, Oshima RG, Adamson ED (1995). Targeted disruption of vinculin genes in F9 and embryonic stem cells changes cell morphology, adhesion, and locomotion. Proc Nat Acad Sci U S A.

[59] Mierke CT, Kollmannsberger P, Zitterbart DP, Diez G, Koch TM, Marg S, Ziegler WH, Goldmann WH, Fabry B (2010). Vinculin facilitates cell invasion into three-dimensional collagen matrices. J Biol Chem.

[60] Mierke CT, Rosel D, Fabry B, Brabek J (2008). Contractile forces in tumor cell migration. Eur J Cell Biol.

[61] Kraning-Rush CM, Califano JP, Reinhart-King CA (2012). Cellular traction stresses increase with increasing metastatic potential. PLoS One.

[62] Liu M, Oberg K, Zhou Y (2007). Expression and function of vinculin in neuroendocrine tumors. Tum Biol.

[63] Ziegler WH, Liddington RC, Critchley DR (2006). The structure and regulation of vinculin. Trends Cell Biol.

[64] Lane J, Martin TA, Watkins G, Mansel RE, Jiang WG (2008). The expression and prognostic value of ROCK I and ROCK II and their role in human breast cancer. Int J Oncol.

[65] Liu X, Choy E, Hornicek FJ, Yang S, Yang C, Harmon D, Mankin H, Duan Z (2011). ROCK1 as a potential therapeutic target in osteosarcoma. J Ortho Res.

[66] Wong CC, Wong CM, Tung EK, Man K, Ng IO (2009). Rho-kinase 2 is frequently overexpressed in hepatocellular carcinoma and involved in tumor invasion. Hepatology.

[67] Kamai T, Tsujii T, Arai K, Takagi K, Asami H, Ito Y, Oshima H (2003). Significant association of Rho/ROCK pathway with invasion and metastasis of bladder cancer. Clin Cancer Res.

[68] Vishnubhotla R, Sun S, Huq J, Bulic M, Ramesh A, Guzman G, Cho M, Glover SC (2007). ROCK-II mediates colon cancer invasion via regulation of MMP-2 and MMP-13 at the site of invadopodia as revealed by multiphoton imaging. Lab Invest.

[69] Anaya J (2016). OncoLnc: linking TCGA survival data to mRNAs, miRNAs, and IncRNAs. PeerJ Computer Sciences.

