# Supplementary material for: Suppression of focal adhesion formation may account for the suppression of cell migration, invasion and growth of non-small cell lung cancer cells following treatment with polyisoprenylated cysteinyl amide inhibitors
Source: Oncotarget. 2018 May 25;9(40):25781–95. doi: 10.18632/oncotarget.25372 (PMC5995249; doi:10.18632/oncotarget.25372)
Supplement: Supplementary file 1 [file oncotarget-09-25781-s001.pdf]

Suppression of focal adhesion formation may account for the suppression of cell migration, invasion and growth of non-small cell lung cancer cells following treatment with polyisoprenylated cysteinyl amide inhibitors

SUPPLEMENTARY MATERIALS

Name	Structure
Polyisoprenylated cellular protein	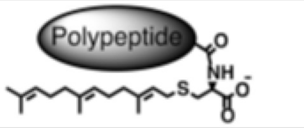
NSL-BA-040	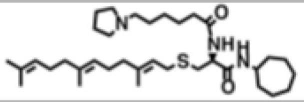
NSL-BA-055	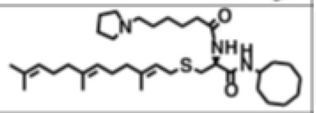
NSL-100	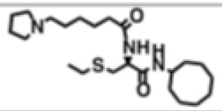

Supplementary Figure 1: Chemical structures of the PCAIs and their non-farnesylated analog, NSL-100. PCAIs (NSL-BA-040, NSL-BA-055) were synthesized to mimic endogenous polyisoprenylated small GTPases such as the polyisoprenylated Rho proteins. Their non-farnesylated analog, NSL-100, lacks the farnesyl moiety that is present in the PCAIs.

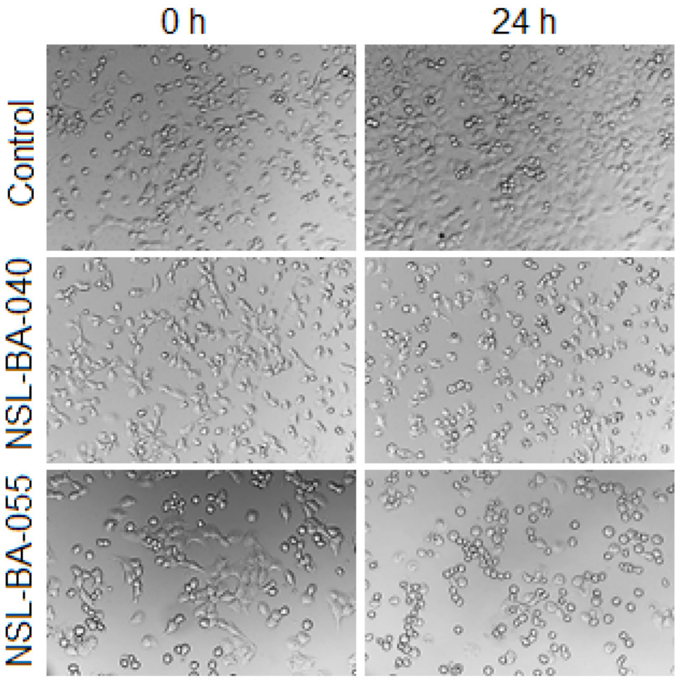

Supplementary Figure 2: PCAIs induce rounding of adherent cells. Images of H1299 cells that were plated in 100 mm tissue culture dishes, exposed to 5 μM of the PCAIs for 24 h. Images were captured at 0 h and 24 h post exposure to the PCAIs using a Nikon Ti Eclipse Microscope.
